# Development of Injectable Calcium Sulfate and Self-Setting Calcium Phosphate Composite Bone Graft Materials for Minimally Invasive Surgery

**DOI:** 10.3390/ijms23147590

**Published:** 2022-07-08

**Authors:** Yu-Hsun Chiu, I-Cheng Chen, Chen-Ying Su, Hsin-Hua Tsai, Tai-Horng Young, Hsu-Wei Fang

**Affiliations:** 1 Institute of Biomedical Engineering, College of Medicine and College of Engineering, National Taiwan University, Taipei 10617, Taiwan; nosebook.tw@gmail.com; 2 Shin Kong Wu Ho-Su Memorial Hospital, Taipei 11101, Taiwan; 3 Accelerator for Happiness and Health Industry, National Taipei University of Technology, No. 1, Sec. 3, Zhongxiao E. Rd., Taipei 10608, Taiwan; icchen.ntut@mail.ntut.edu.tw; 4 Department of Chemical Engineering and Biotechnology, National Taipei University of Technology, No. 1, Sec. 3, Zhongxiao E. Rd., Taipei 10608, Taiwan; chenying.su@gmail.com (C.-Y.S.); m6023313@gmail.com (H.-H.T.); 5 Institute of Biomedical Engineering and Nanomedicine, National Health Research Institutes, Miaoli City 35053, Taiwan

**Keywords:** injectable, biomaterials, calcium sulfate hemihydrate, calcium phosphate, minimally invasive surgery

## Abstract

The demand of bone grafting is increasing as the population ages worldwide. Although bone graft materials have been extensively developed over the decades, only a few injectable bone grafts are clinically available and none of them can be extruded from 18G needles. To overcome the existing treatment limitations, the aim of this study is to develop ideal injectable implants from biomaterials for minimally invasive surgery. An injectable composite bone graft containing calcium sulfate hemihydrate, tetracalcium phosphate, and anhydrous calcium hydrogen phosphate (CSH/CaP paste) was prepared with different CSH/CaP ratios and different concentrations of additives. The setting time, injectability, mechanical properties, and biocompatibility were evaluated. The developed injectable CSH/CaP paste (CSH/CaP 1:1 supplemented with 6% citric acid and 2% HPMC) presented good handling properties, great biocompatibility, and adequate mechanical strength. Furthermore, the paste was demonstrated to be extruded from a syringe equipped with 18G needles and exerted a great potential for minimally invasive surgery. The developed injectable implants with tissue repairing potentials will provide an ideal therapeutic strategy for minimally invasive surgery to apply in the treatment of maxillofacial defects, certain indications in the spine, inferior turbinate for empty nose syndrome (ENS), or reconstructive rhinoplasty.

## 1. Introduction

Bone grafting is one of the most commonly used surgical methods for bone regeneration since bone defects resulting from diseases, trauma, or surgery have become a significant health issue globally. Over two million bone grafting procedures are performed annually worldwide, and the demand is dramatically increasing as population ageing is a serious problem in many countries [1,2]. Although an autologous bone graft is still considered as the gold standard in terms of better biocompatibility, high bioactivity, and non-immunogenicity, limited availability and donor site morbidity are the major concerns.

Since the risks of disease transmission and an immunogenic response are downsides of allografts and xenografts, there has been great interest in the use of synthetic biomaterials as bone graft implants [3]. Degradable materials such as bioceramics are alternatives to avoid donor site injury and superior for bone repairs due to their improved biocompatibility, osteoconductivity, consistent material properties, and low cost. Among them, calcium sulfate (CS) and calcium phosphate (CaP) have demonstrated the ability to partially integrate into bone tissue and stimulate osteoblast growth and have been widely used in the place of allografts or autografts for several decades [4,5,6,7,8,9]. CS possesses several desirable properties including a low curing temperature, rapid setting, biocompatibility, biodegradability, and promotion of bone healing. Nevertheless, the fast resorption of CS before new bone formation may result in gaps and negatively affect its clinical outcome. CaPs are biocompatible, biodegradable, and highly porous materials that possess similarity in structure to the inorganic composition of bone minerals, and under in vivo conditions it can transform to hydroxyapatite (HA), which is the dominant mineral phase of vertebral tooth and bone tissue. Although CaP exerts osteoconductivity to achieve bone regeneration, it typically lacks mechanical stability, which limits its clinical applications [10].

Although bone graft materials have been extensively developed over the decades, only a few injectable bone grafts are clinically available and none of them can be extruded from 18G needles [11,12]. An ideal bone graft for minimally invasive surgery should have a good injectability, a controlled working time/setting time, and an ease of handling and delivery. In this study, we prepared a novel bone graft substitute by combining the properties of CS and CaP as a composite bone graft with great injectability, adequate handling, and mechanical properties that can be used for further development for performing minimally invasive surgery.

## 2. Results

### 2.1. The Synthesis of Calcium Sulfate Hemihydrate (CSH, CaSO_4_·0.5H_2_O)

**Figure 1:**
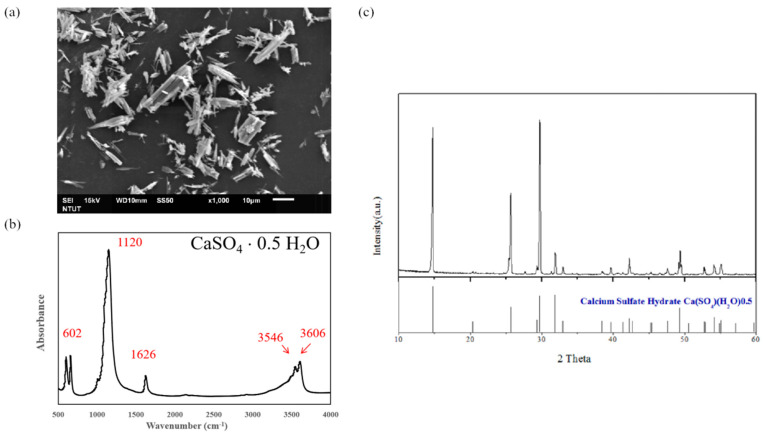
Characterization of synthesized CSH: (**a**) SEM image of CSH shows the rod-like morphology of CSH; (**b**) The FTIR spectra presents the typical profiles corresponding to the CSH structure; and (**c**) XRD analysis reveals the synthesis of CSH from CSD phase.

CS has been approved by the U.S. Food and Drug Administration (FDA) as a bone graft material due to its non-toxicity, superb workability, good biocompatibility, and osteoconductivity, and the hemihydrate form of CS (calcium sulfate hemihydrate, CSH, CaSO_4_·0.5H_2_O) is known to be suitable for clinical applications such as a bone void filler and is widely used in orthopedics and dentistry [5,9,13,14,15,16]. In our previous studies, we have developed a hydrothermal method to produce self-prepared CSH by high vapor pressure of water [17,18]. Figure 1a shows the rod-like morphology of CSH observed by scanning electronic microscopy (SEM) and the size of the CSH specimen is smaller than 40 μm. CSH underwent Fourier transform infrared spectroscopy (FTIR) analysis to determine the functional groups and the results of the FTIR spectra are displayed in Figure 1b, indicating typical profiles corresponding to the CSH structure. X-ray diffraction (XRD) analysis was conducted to evaluate the conversion of the calcium sulphate dihydrate (CSD) phase to the CSH phase after heating in CaCl_2_ solution, and all of the corresponding XRD peaks were analyzed according to the Joint Committee on Powder Diffraction Standards (JCPDS). Figure 1c presents the typical CSH XRD peaks, revealing the presence and the synthesis of CSH from the CSD phase [19]. The homemade CSH was further used to produce composite bone materials.

### 2.2. Altering the Handling Property of CSH/CaP Composite Bone Materials by Citric Acid

The fast resorption of CSH before new bone formation is the main limitation of this material and it may result in gaps and negatively affect its clinical outcome for the regeneration of bone. CaPs are biocompatible, biodegradable, and highly porous material that possess a similarity in structure to the inorganic composition of bone minerals, exerting osteoconductivity to achieve bone regeneration, but typically lack mechanical stability [10]. Basic CaP cement is relatively stable chemically under physiological conditions and may still be present even after years in vivo. In this study, we prepared a novel injectable bone graft substitute by combining the properties of CSH and CaP (tetracalcium phosphate and dicalcium phosphate anhydrous; TTCP and DCPA) as a composite bone graft for minimally invasive surgery. TTCP (Ca_4_(PO_4_)_2_O) was selected because it contains a higher Ca/P ratio and forms HA under physiological conditions when combined with an acidic calcium phosphate such as DCPA (CaHPO_4_) [20].

**Figure 2:**
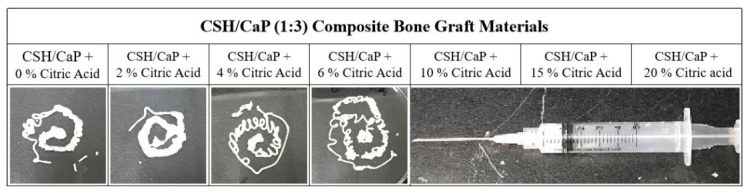
The images show injectability of CSH/CaP (1:3) bone graft materials containing different concentrations of citric acid tested by 18G syringe injection. The materials lost injectability when concentrations of citric acid were 10%, 15% and 20%.

To prepare injectable bone graft materials, CSH and CaP (equal amount of TTCP and DCPA) were firstly combined in a 1:3 ratio as powder components and then mixed with liquid to form a paste, which can be delivered into defects by injection. Although this paste could be fully injected from the syringe equipped with 18G needles, the setting time was longer than 120 min (Table 1, Figure 2). Additives such as citric acid are known to change the setting reaction and improve the injectability of CaP cements [21,22], thus, various concentrations (2~20%) of citric acid were added into the powder component to prepare bone graft materials. As Table 1 shows, the setting time decreased greatly with an increased concentration of citric acid. When the bone materials were supplemented with 4% to 20% of citric acid, the setting time was reduced, ranging from 68 min to 31 min. Figure 2 presents the influence of the content of citric acid on the injectability of the CSH/CaP pastes and the results indicated that the materials lost injectability when concentrations of citric acid were 10%, 15%, and 20% (Figure 2).

### 2.3. Effects of CSH/CaP Ratio on Mechanical Properties

**Figure 3:**
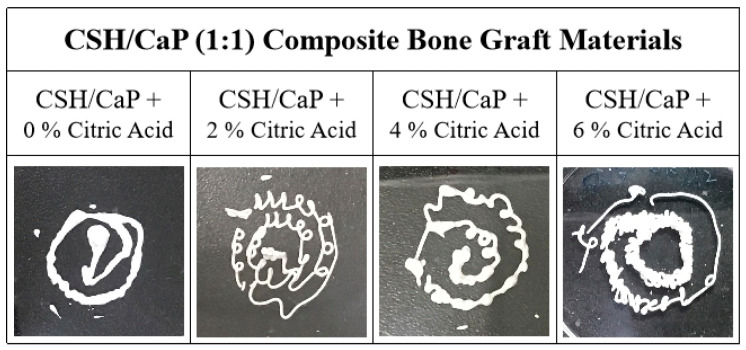
Injectability tests of CSH/CaP (1:1) bone graft materials containing different concentrations of citric acid show that all test samples can be extruded by 18G syringe injection.

**Figure 4:**
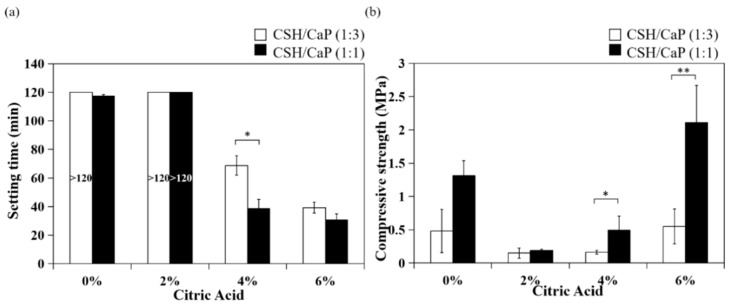
Effects of CSH/CaP ratios (1:3 white bars; 1:1 black bars) on the properties of CSH/CaP composite graft materials: (**a**) comparison of handling property–setting time of test samples; (**b**) comparison of mechanical property–compressive strength of test samples. * *p* < 0.05 and ** *p* < 0.01 when compared with CSH/CaP (1:3) samples.

Next, bone materials with combining equimolar amounts of CSH and CaP (1:1) were prepared as presented in Table 2, and the setting and mechanical properties were examined and compared with the CSH/CaP (1:3) composite. Four groups were classified based on the percent of citric acid (0, 2, 4, and 6%) and all groups were injectable from 18G needles (Figure 3). The setting time was greatly reduced with the addition of citric acid (4% and 6%, 39 min and 31 min, Table 2). Interestingly, the setting time of the CSH/CaP (1:1) group (Figure 4a, black bar) was significantly shorter than the CSH/CaP (1:3) group (Figure 4a, white bar) when the concentration of citric acid was 4% (39 min vs. 68 min, *p* = 0.044). The mechanical properties of CSH/CaP paste were tested by a universal mechanical testing machine. As shown in Figure 4b, the compressive strength was also significantly higher when the CSH/CaP ratio was 1:1 supplemented with 4% or 6% of citric acid (4% citric acid, CSH/CaP 1:3 vs. 1:1, 0.16 vs. 0.49 MPa, *p* = 0.024; 6% citric acid, CSH/CaP 1:3 vs. 1:1, 0.55 vs. 2.11 MPa, *p* = 0.001). Altogether, CSH/CaP at a ratio of 1:1 was selected for further optimization of the bone materials.

### 2.4. Effects of Hydroxyl Propyl methyl Cellulose (HPMC) on the Properties CSH/CaP Composite Bone Graft Materials

**Figure 5:**
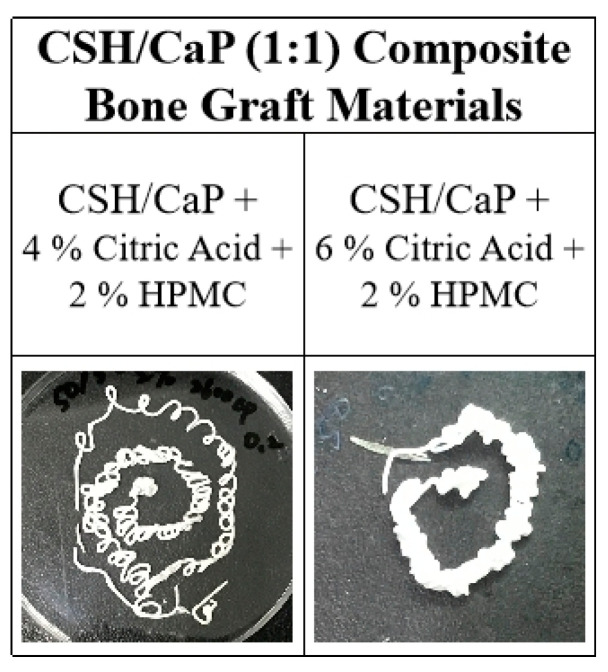
Injectability tests of CSH/CaP (1:1) bone graft materials containing different concentrations of citric acid and 2% HPMC show that all test samples can be extruded by 18G syringe injection.

**Figure 6:**
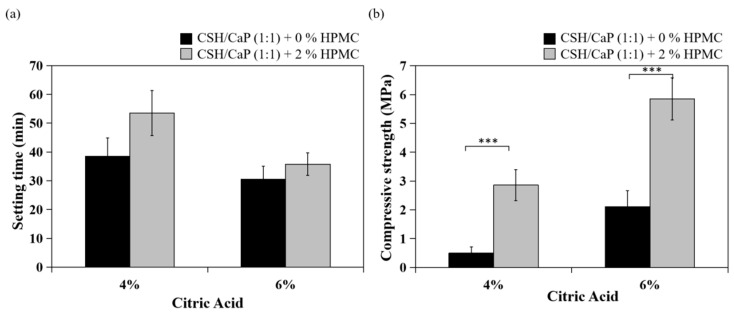
Effects of HPMC (0% black bars; 2% gray bars) on the properties of CSH/CaP (1:1) composite graft materials: (**a**) comparison of handling property–setting time of test samples; (**b**) comparison of mechanical property–compressive strength of test samples. *** *p* < 0.001 when compared with CSH/CaP (1:1) + 0% HPMC samples.

The CaP cement is vulnerable to wash out before hardening occurs. The presence of additives such as hydroxyl propyl methyl cellulose (HPMC) was previously shown to affect handling properties, washout resistance, cement hardening behavior, and mechanical properties of calcium phosphate cements [18,23]; thus, 2% of HPMC was added to the powder phase of CSH/CaP (1:1) bone materials and examined (Table 3). The setting time was 54 min or 36 min when CSH/CaP (1:1) bone materials were supplemented with 2% HPMC contained 4% citric acid or 6% citric acid, respectively (Table 3). As shown in Figure 5, both HPMC added groups showed injectability with either 4% or 6% citric acid. In terms of HPMC content, the setting time was not changed when it was increased from 0 to 2% (Figure 6a). By contrast, the addition of 2% HPMC significantly enhanced the compressive strength of the CSH/CaP bone materials (Figure 6b; 4% citric acid, 0% HPMC vs. 2% HPMC, 0.49 vs. 2.86 MPa, *p* < 0.001; 6% citric acid, 0% HPMC vs. 2% HPMC, 2.11 vs. 5.85 MPa, *p* < 0.001).

**Figure 7:**
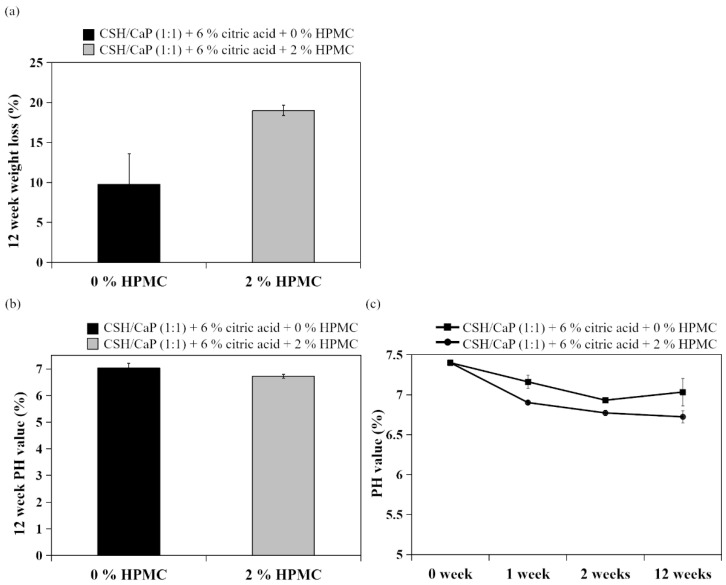
Comparison of CSH/CaP (1:1) composite graft materials without (black bars) or with 2% HPMC (gray bars): (**a**) degradation test of test samples; (**b**,**c**) pH value measurement of test samples at weeks 0, 1, 2, and 12.

To further investigate the washout resistant property from HPMC addition, cement specimens were disintegrated in PBS with agitation for 12 weeks. Surprisingly, from the degradation results in Figure 7a, it can be seen that the addition of HPMC did not statistically alter the weight loss of specimens. The pH values of specimens were dropped from 7.4 (week 0) to around 6.6–7.1 (week 12) and showed no significant difference with or without HPMC (Figure 7b,c).

**Figure 8:**
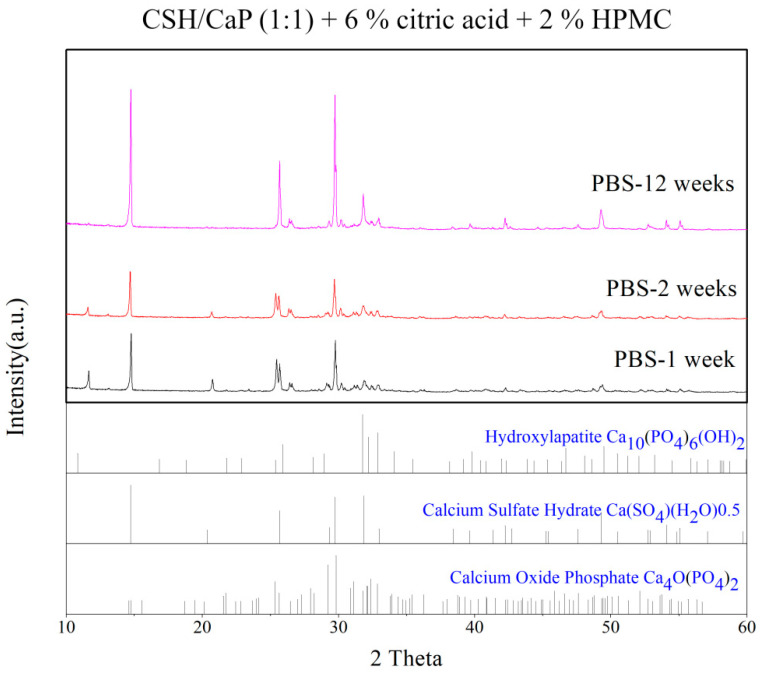
XRD profiles of CSH/CaP paste (CSH/CaP 1:1, 6% citric acid, 2% HPMC) immersed in PBS for 1, 2, and 12 weeks presented that the HAp peaks appeared at week 12.

In general, CaP cement sets in situ with an intimate adaptation to the defect surfaces and forms an implant similar to hydroxyapatite. Therefore, the samples fabricated with equimolar amounts of CSH and CaP (1:1), 6% citric acid and 2% of HPMC were tested by the XRD method to determine the extent of chemical conversion. Figure 8 presented that the HAp peaks appeared in the XRD profiles through incubation for 12 weeks, indicating the transformation of TTCP and DCPD to HAp.

### 2.5. In Vitro Cytotoxicity of CSH/CaP Paste

**Figure 9:**
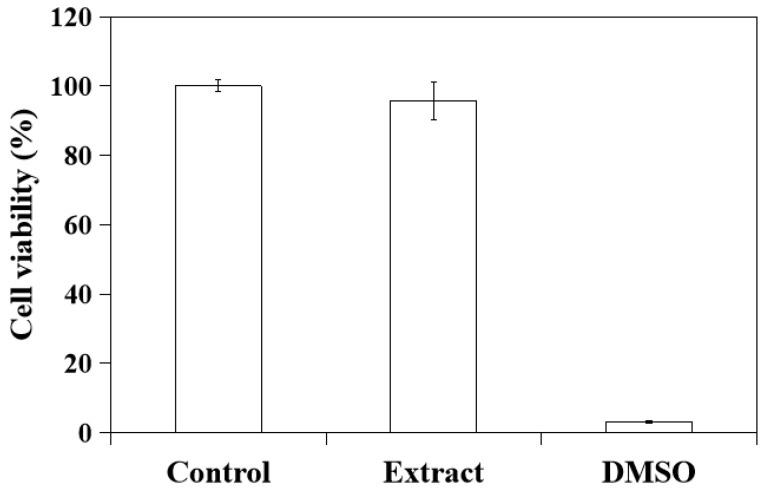
Cell viability of L929 cells measured by MTT assay for evaluating the cytotoxicity of CSH/CaP paste (CSH/CaP 1:1, 6% citric acid, 2% HPMC). Control: viability of L929 cells cultured in regular medium, set as 100% (blank); Extract: viability of L929 cells cultured in medium containing bone material extract; DMSO: viability of L929 cells cultured in medium containing 10% DMSO (negative control).

To evaluate the cell toxicity of CSH/CaP composite paste (CSH/CaP 1:1, 6% citric acid, 2% HPMC), the specimen was immersed in culture medium and the extract from the bone material was used for culturing with L929 cells for the MTT test. As Figure 9 shows, the average cell viability from the extract group was 95.73% (>70%), indicating the CSH/CaP composite paste shall be considered non-cytotoxic for future applications.

## 3. Discussion

One initial objective of this study was to create an injectable and moldable bone graft from synthetic materials in place of an autograft to avoid invasive surgical procedures and eliminate donor site morbidity caused by harvesting autografts, especially for minimally invasive surgeries to treat maxillofacial defects or empty nose syndrome (ENS). We utilized CSH and CaP as biomaterials to obtain an ideal composite product with biocompatible, degradable, and osteoconductive properties. The advantage of this material is that it can be adjusted to the target place with different shapes by a minimally invasive procedure.

### 3.1. Effects of Citric Acid on Setting Time and Injectability

To control the setting process of CaP cements, several parameters including the particle size, the composition of the cement reactants, and the additives could be adjusted to alter their handling and mechanical properties. As Table 1 and Figure 4 and Figure 6 show, supplementing with citric acid reduced the working/setting time and improved the compressive strength of CSH/CaP composite materials. However, the bone graft materials lost their injectability when the concentration of citric acid was higher than 10%. Citric acid was proposed to act as a water-reducing agent [21]. Our results match those mentioned in earlier studies that adding sodium salts of the oligocarboxylic acids (malic, tartaric, and citric acids) with optimal concentrations to CaP cement caused a liquefying effect combined with an enhancement of the mechanical strength. It was also hypothesized that citric acid could improve the injectability and strength of apatite cements because of the reduced porosity or homogeneous and denser microstructure of CaP cement that worked as the base of growth and entanglement of apatite crystals [21,24,25]. It is worth mentioning that the paste developed in this study possesses a great injectability such that it could be extruded through 18G needles, differing from other published injectable pastes [11,25,26,27].

### 3.2. Effects of the Ratio of CSH and CaP

In addition to citric acid, the trends noted in Figure 4b presented that the mixture ratio of CSH/CaP also influenced the mechanical capacity of the materials. There was a significant positive correlation between the compressive strength and the ratio of CSH in the mixture. This finding suggested that while the lack of mechanical strength is a drawback of CaP, CSH could be a good compatible material to offer further improvements to their mechanical properties and wider applications [28,29,30].

### 3.3. Effects of HPMC on Anti-Washout Ability and Mechanical Property

Numerous biopolymers, such as sodium alginate, HPMC, hyaluronic acid, chitosan, and modified starch have been reported to increase the viscosity of bone grafts to improve the cohesion and anti-washout ability [31,32,33,34,35,36]. As mentioned in the reports above, small amounts of these biopolymers could notably improve the cohesion and anti-washout of CaP cements. Evidence showed that that the handling properties were greatly improved by the addition of gelling agents such as HPMC, carboxyl methylcellulose (CMC), and chitosan in TTCP and DCPA [33]. The addition of 1% HPMC notably increased the paste injectability of dicalcium phosphate dihydrate [11]. HPMC is one of the most commonly occurring polysaccharides derived from alkali-treated cellulose and is often used as a gelling agent. In terms of HPMC content, our previous study showed that an increased proportion of HPMC exerted an anti-washout property of calcium sulfate/calcium phosphate premixed putty, leading to less decay and larger disintegrating particles [18]. In this study, 2% of HPMC as a polymer carrier was expected to function as a matrix, increase the viscosity of the paste, and improve the washout resistance of bone cement. Surprisingly, the addition of 2% HPMC was found to have no significant effect on the degradation of CSH/CaP paste. Despite the obscure effect of the anti-washout property in this study, HPMC indeed enhanced the mechanical strength (Figure 6b), which might be determined by its microstructure. The relationship between microstructure and mechanical property in this study is worth further investigation. In addition, an increase in the compressive strength of the CaP cements could be due to the transformation of TTCP and DCPD to Hap, or partial CSD to Hap [25]. Figure 8 provided the evidence that TTCP and DCPD partially transformed to Hap. Animal tests with the injectable paste (CSH/CaP 1:1 supplemented with 6% citric acid and 2% HPMC) will be performed in the near future to further confirm the bone repair effects in vivo.

### 3.4. Possible Clinical Applications

The compressive strength of the injectable paste (CSH/CaP 1:1 supplemented with 6% citric acid and 2% HPMC) in this study was around 5.85 MPa, which is similar to that of human cancellous bone (between 4 and 12 MPa) and is suitable for low load bearing applications such as the treatment of maxillofacial defects, certain indications in the spine, reconstructive rhinoplasty, or inferior turbinate for ENS.

ENS is recognized as an iatrogenic condition, most frequently following aggressive inferior turbinate resection [37,38,39]. The symptoms are most often referred as the presence of paradoxical nasal obstruction despite an objectively patent nasal cavity, a lack of airflow sensation, a feeling of suffocation, nasal dryness, and neuropathic pain [40]. The first line of management includes nasal moisturization and humidification, supportive therapies of concomitant medical conditions (e.g., panic, anxiety, and depression) [41,42], and surgery. Surgical treatment to augment the loss of turbinate volume with temporary fillers, synthetic implants, and autologous materials has been reported to be an effective method [38,40,43]. Nevertheless, the injectable paste developed in this study exerts a better therapeutic potential than the current temporary fillers (e.g., hyaluronic acid), not only in strength but also persistence [25]. Patients could receive submucosal injection of the injectable paste into the remnant inferior turbinate by a 18G spinal needle to increase tissue bulk via endoscopy under local anesthesia. The non-surgical and minimally invasive turbinate augmentation will be a great option for patients diagnosed with ENS who are not suitable for an autologous bone graft, and more patients suffering from ENS can be treated with a very low risk of side effects. The future works will focus on the osteogenic effects by testing the materials with osteogenic cell lines such as human primary osteoblast cells, human osteosarcoma, derived SaOS-2 cells, or the osteoblast cell line MG63. Moreover, a New Zealand rabbit animal model with bone defects will be established for further investigation.

## 4. Materials and Methods

### 4.1. Calcium Sulfate Hemihydrate (CSH) Preparation

The α-form of CSH (CaSO_4_·0.5H_2_O) was prepared by a wet method as described [17]. Briefly, CSD (CaSO_4_·2H_2_O; J.T. Baker, Phillipsburg, NJ, USA) was heated at 132 °C for 30 min, followed by mixing with 30% CaCl_2_ (SHOWA Corporation, Osaka, Japan) solution and the mixture was heated at 132 °C for 30 min again. CaCl_2_ was washed and removed by water, and the rest was incubated at 132 °C for 30 min. After the reaction finished, the resultant product was dried in oven at 50 °C then milled by a high-efficiency ball mill for 4 min (8000 M Mixer, SPEX, Metuchen, NJ, USA).

### 4.2. Scanning Electron Microscope (SEM)

The morphology of synthetic CSH powder were assessed by SEM using an S-3000H microscope (Hitachi, Tokyo, Japan) under low vacuum conditions with an accelerating voltage of 15 kV. Each specimen was covered with gold by a sputter coater (Ion Sputter E101, Hitachi).

### 4.3. Fourier Transform Infrared Spectroscopy (FTIR)

The CSH powder was mixed with KBr salt at a 1:100 ratio and prepared as pellets. The IR spectra of sample pellet were acquired in transmittance mode between the range of 4000 and 500 cm^−1^ and recorded using a FTIR spectrophotometer (Spotlight 200i Sp2, PerkinElmer, MA, USA).

### 4.4. X-ray Diffraction (XRD)

The X-ray diffraction profiles of specimens were recorded by X-ray powder diffraction analysis (x’pert3 powder, PANalytical B.V., Almelo, the Netherlands) to investigate the crystalline phases of synthetic CSH. Cu Kα X-rays were used generated at 45 kV and 40 mA at a diffraction angle (2θ) varied from 10° to 60° with a step size of 0.1°/step and an interval of 0.2 s/step. The phase composition was checked by means of JCPDS reference patterns.

### 4.5. CSH/CaP Composite Bone Graft Formulation and Paste Preparation

The composite bone graft materials contained a powder and liquid phase. The basic powder component was a mixture of CSH and CaP. CaP component consisted of TTCP (Ca_4_(PO_4_)_2_O) and DCPA (CaHPO_4_) (Alfa Aesar, Lancashire, UK) in a 1:1 molar ratio. Homemade CSH powder was then mixed with CaP component (in the ratios of 1:1 or 1:3) and different concentrations of citric acid and HPMC (both from Sigma, St. Louis, MO, USA). Distilled water was added as liquid phase and mixed with powder phase described above to produce paste for further experiments.

### 4.6. Setting Time Measurement

The self-setting time of prepared CSH/CaP composite bone graft materials was tested according to the ASTM 266 standard and measured using a Gilmore needle test and evaluated every 30 s at room temperature (22 ± 1 °C). Working time (initial setting time) was defined when 0.3 MPa was loaded onto the specimen using a needle with no visible marks on the surface, while 5 MPa was used for evaluating setting time (final setting time).

### 4.7. Injectability

The injectability of each prepared sample was monitored by whether the paste could be gently extruded from a 5 mL disposable syringe equipped with an 18G needle.

### 4.8. Mechanical Compression Testing

To prepare the samples for the compressive strength test, the powder component and liquid component were mixed under atmospheric conditions at room temperature and spatulated into stainless steel molds to make cylindrical shapes with a diameter of 6 mm and a height of 12 mm for the mechanical test. The compressive strength of each sample was examined in accordance with ASTM F1633 standards measured at a loading rate of 1 mm/min with a universal mechanical testing machine (Transcell Technology Inc., New Taipei City, Taiwan). The measurements were performed three times for each group.

### 4.9. Degradation Test and pH Value Measurement

The prepared CSH/CaP pastes were molded into columns measuring 6 mm in diameter and 3 mm in height and transferred into phosphate-buffer saline (PBS pH 7.4, Gibco, Thermo Fisher Scientific Inc., Waltham, MA, USA) in an incubator at 37 °C for different time period. The weight loss ratio (washout) was calculated from the formula of the weight loss of the specimen divided by the weight of initial sample. The pH value for each sample was obtained at week 1, 2, and 12 from PBS. Each test was repeated for three times and the average value was calculated.

### 4.10. Cell Viability

To conduct cell viability assay for medical device, L929 cells (mouse fibroblasts, strain number BCRC 60091) were suggested by ISO10993-5 standard [44]. L929 cell line was purchased from the Food Industry Research and Development Institute, Hsinchu, Taiwan. Cells were routinely maintained in Modified Eagle Medium (MEM, Gibco, Thermo Fisher Scientific Inc., Waltham, MA, USA) supplemented with 10% fetal bovine serum (FBS, Invitrogen, Waltham, MA, USA) at 37 °C under 5% CO_2_ and 95% relative humidity.

To evaluate biocompatibility of bone materials, test samples (CSH/CaP 1:1, 6% citric acid, 2% HPMC) were firstly extracted following ISO10993-12 standard [45]. Two grams of test sample was extracted in 10 mL of MEM/10% FBS (extraction ratio 0.2 g/mL) at 37 °C for 24 h and the medium was collected for the treatment of cell viability test. Cell viability was performed according to ISO10993-5 standard [44]. L929 cells were plated in a density of 1 × 10^4^ cells/well onto a 96-well culture plate in MEM/10% FBS at 37 °C overnight. On the next day, culture medium was removed, cells were washed with phosphate-buffer saline (PBS) and cultured in (1) bone material extract described above; (2) MEM/10% FBS (blank); or (3) MEM/10% FBS containing 10% DMSO (negative control, Sigma, St. Louis, MO, USA) for 24 h (*n* = 3 for each treatment). In vitro cell viability test was performed by 3-(4,5-dimethylthiazol-2-yl)-2,5-diphenyltetrazolium bromide method (MTT). MTT solution (Sigma, St. Louis, MO, USA) was added into the medium and cells were incubated at 37 °C for 2 h. The samples were read by an enzyme-linked immunosorbent assay (ELISA) reader (Tecan, Männedorf, Switzerland) with a wavelength of 570 nm to obtain OD values. The viability of L929 cells cultured in MEM/10% FBS without bone material extract (blank) was set as 100% for the assay. Cell viability higher than 70% was considered non-cytotoxic.

### 4.11. Statistics

All the data are represented as mean ± SD of three independent experiments. Statistical difference was evaluated by two-tailed Student’s *t*-test. A value of *p* < 0.05 was considered to be statistically significant.

## 5. Conclusions

In this study, we developed an injectable and moldable bone graft from CSH/CaP composite materials with good handling properties, great biocompatibility, and adequate mechanical strength. Moreover, we demonstrated that the paste could be extruded from a syringe equipped with 18G needles and exerted a great potential for minimally invasive surgery. We strongly believe that this study offers useful information for developing and manufacturing injectable bone graft materials for treating maxillofacial defects, certain indications in the spine, inferior turbinate for ENS, or reconstructive rhinoplasty.

## Figures and Tables

**Table 1:** Chemical compositions and handling properties of CSH/CaP (1:3) bone graft materials.

CSH/CaP (1:3) Composite Bone Graft Materials							
Powder component	CSH/CaP + 0% Citric Acid	CSH/CaP + 2% Citric Acid	CSH/CaP + 4% Citric Acid	CSH/CaP + 6% Citric Acid	CSH/CaP + 10% Citric Acid	CSH/CaP + 15% Citric Acid	CSH/CaP + 20% Citric acid
L/P	0.2	0.2	0.2	0.25	0.25	0.3	0.4
Working time	97 min	64 min	16 min	15 min	15 min	10 min	8 min
Setting time	>120 min	>120 min	68 min	39 min	>60 min	31 min	40 min
Injectability	Yes	Yes	Yes	Yes	No	No	No

CSH: Calcium sulfate hemihydrate; CaP: calcium phosphate; L/P: Liquid/Powder ratio.

**Table 2:** Chemical compositions and handling properties of CSH/CaP (1:1) bone graft materials.

CSH/CaP (1:1) Composite Bone Graft Materials				
Powder component	CSH/CaP + 0% Citric Acid	CSH/CaP + 2% Citric Acid	CSH/CaP + 4% Citric Acid	CSH/CaP + 6% Citric Acid
L/P	0.2	0.2	0.2	0.2
Working time	61 min	50 min	23 min	15 min
Setting time	117 min	>120 min	39 min	31 min
Injectability	Yes	Yes	Yes	Yes

**Table 3:** Chemical compositions and handling properties of CSH/CaP (1:1) bone graft materials.

CSH/CaP (1:1) Composite Bone Graft Materials		
Powder component	CSH/CaP (1:1) + 4% Citric Acid + 2% HPMC	CSH/CaP (1:1) + 6% Citric Acid + 2% HPMC
L/P	0.2	0.25
Working time	11 min	10 min
Setting time	54 min	36 min
Injectability	Yes	Yes

## Data Availability

The data presented in this study are available on request from the corresponding author.
